# Complete Genome Sequences of Two Human Respiratory Syncytial Virus Genotype A Strains from India, RSV-A/NIV1114046/11 and RSV-A/NIV1114073/11

**DOI:** 10.1128/genomeA.00165-13

**Published:** 2013-07-25

**Authors:** Manohar Lal Choudhary, Bhumika S. Wadhwa, Santosh M. Jadhav, Mandeep S. Chadha

**Affiliations:** Human Influenza Department, National Institute of Virology, Pune, India

## Abstract

Two complete genomes of human respiratory syncytial virus subtype A (HRSV-A), with and without a 72-nucleotide duplication in the C-terminal glycoprotein G gene, were sequenced and analyzed. Characterization of these genomes will improve understanding of the diversity, emergence, virulence, pathogenicity, and transmissibility of a novel RSV-A genotype with a 72-nucleotide G gene duplication.

## GENOME ANNOUNCEMENT

Human respiratory syncytial virus (HRSV) is a major cause of lower respiratory tract infection (LRTI) in neonates, children, and elderly patients (1). It causes repeated infections throughout life due to limited immune protection from earlier exposure (2). This virus belongs to the *Pneumovirus* genus within the family *Paramyxoviridae*. HRSV is an enveloped, negative-sense, single-stranded RNA genome of 15 kb that encodes 11 viral proteins. RSV is divided into two antigenic subgroups, A and B, on the basis of the reactivity of the virus with monoclonal antibodies against the attachment (G) and fusion (F) glycoproteins (3). To date, 10 HRSV-A genotypes have been designated, GA1 to GA7, SAA1, NA1, and NA2. The HRSV-B genotypes include GB1 to GB4, SAB1 to SAB3, and BA1 to BA6 (4, 5). A 60-nucleotide duplication in the C terminus of the G gene in RSV-B was identified in South America (Argentina) in 1999 and is now circulating worldwide (4). Recent reports from Canada and Korea have identified a novel 72-nucleotide duplication in the C-terminal part of the glycoprotein G gene in RSV-A (6, 7).

In this study, the complete genomes of two newly emerged HRSV-A strains, one (RSV-A/NIV1114046/11) with a 72-nucleotide duplication in the region of the G gene corresponding to the C terminus and one (RSV-A/NIV1114073/11) without the duplication, have been determined directly from clinical specimens. The full-length RSV-A genome was amplified as 15 overlapping PCR fragments, as described earlier (8), using a superscript One Step reverse transcription (RT)-PCR kit (Invitrogen). Complete sequences were obtained by use of fragment-specific and inner primers using an ABI 3730xl DNA sequencer (Applied Biosystems, Foster City, CA). The obtained sequences were analyzed by sequence analysis software and alignment was done using DAMBE and MEGA5 software.

The RSV-A/NIV1114046/11 genome was 15,231 bp in length, including a 3′ leader, a 5′ trailer, and a 72-nucleotide duplication in the region of the G gene corresponding to the C terminus. The RSV-A/NIV1114073/11 genome was 15,176 bp in length, including a 3′ leader and 5′ trailer. From these, open reading frames (ORFs) of 11 encoded viral proteins were deduced for the NS1, NS2, N, M, P, G, F, SH, M2-1, M2-2, and L genes. The nucleotide sequence variability of HRSV-A ranged from 0.36% to 5.21% compared with database whole-genome information for HRSV-A (7–9). The deduced amino acid sequence identity ranged from 95.47% to 99.72%, in accordance with the ORFs. Overall G+C content was 33.46%, and the smallest GC ratio was observed in the M2-2 gene (28.83% for RSV-A/NIV1114046/11 and 28.46% for RSV-A/NIV1114073/11).

Phylogenetic analysis showed that the RSV-A/NIV1114046/11 strain, with the 72-nucleotide duplication in the G gene, clustered together with the HRSV-A/GN435/11 and HRSV-A/ON1 strains from Korea and Canada (6, 7) and RSV-A/NIV1114073/11 clustered with A/WI/629-Q0284/10 from the United States (9).

### Nucleotide sequence accession numbers.

The complete genome sequences of the novel RSV-A/NIV1114046/11 and RSV-A/NIV1114073/11 strains were submitted to GenBank and assigned accession numbers KC731482 and KC731483, respectively.
